# Exploration of Concerns about the Evidence-Based Guideline Approach in Conservation Management: Hints from Medical Practice

**DOI:** 10.1007/s00267-020-01312-6

**Published:** 2020-06-27

**Authors:** Fabian Gutzat, Carsten F. Dormann

**Affiliations:** Department of Biometry and Environmental System Analysis, University of Freiburg, Tennenbacher Str. 4, 79106 Freiburg, Germany

**Keywords:** Evidence-based guidelines, Evidence-based practice, Decision support, Biodiversity conservation, Implementation gap

## Abstract

The importance of using evidence in decision-making is frequently highlighted in policy reports and scientific papers. However, subjective judgments of the reliability of environmental evidence vary widely, and large-scale systematic searches for evidence are only common for climate-related topics. In the medical field, evidence-based guidelines are routinely used to guide treatments. In the management of multiple-use landscapes similar guidelines could substantially narrow the science-practice gap but are largely absent. The challenges potential guidelines face are therefore unknown. For the case of forest conservation, we conducted 14 semistructured interviews with mainly forest practitioners and presented them an example medical guideline together with evidence-based statements on forest conservation (hereinafter: statement paper). We identified 28 concerns related to potential evidence-based guidelines in forest conservation. The interviews yielded approximately three major findings. First, recommendations on forest conservation are better accepted if they include clear instructions and are formulated for a specific context. Fragmentary conservation evidence complicates the formulation of specific recommendations. Second, the level of evidence framework, which indicates the strength of the available evidence, is perceived as too complex. Third, neglecting forest multifunctionality in a potential guideline hampers its application but, if addressed, potentially weakens its ecological relevance. We show that major concerns about potential evidence-based conservation guidelines are similar to the challenges experienced by medical guidelines. We also identify concerns unique to forestry.

## Introduction

The importance of evidence-based policy-making is emphasized in countries around the globe (e.g., Russell-Smith et al. 2015; Cooke et al. 2016; Majcen 2017). With the release of the Intergovernmental Panel on Climate Change (2019) reports, climate-related policy-makers and the general public can access systematically searched and assessed scientific literature since decades. In other fields of environmental management, such as biodiversity conservation, similar reports with synthesized and assessed scientific evidence are still emerging (Intergovernmental Science-Policy Platform on Biodiversity and Ecosystem Services 2019). Consequently, on-ground management frequently relies on weak evidence (such as anecdotes, Sutherland and Wordley 2017).

Forests provide multiple ecosystem services (e.g., timber, recreation, carbon storage) to different stakeholders (Primmer and Kyllönen 2006; Schaich and Plieninger 2013; Trivino et al. 2017; St-Laurent et al. 2018). A variety of interests related to the preferred land use, as in the case of forests, can make decision-making irreproducible and opaque to the public (e.g., Adams and Sandbrook 2013; Bainbridge 2014; Donnelly et al. 2018). A lack of transparency provides the breeding ground for more influential stakeholders to compromise decision-making, even if evidence is available (Juntti et al. 2009).

In medicine, there is a well-established process to guide evidence synthesis, with clear guidance about systematic evidence synthesis, the judgment of evidence reliability, and more transparent decision-making.

Evidence-based practice has been well known for more than two decades among experts from different health disciplines, such as medicine, nursing, and psychology (e.g., Sackett et al. 1996; American Psychological Association 2006; Mackey and Bassendowski 2017). Meta-analyses, systematic reviews, and medical guidelines are essential for evidence-based practice, as they provide synthesized (possibly conflicting) scientific evidence together with implications for practice (Cochrane Collaboration 2019; see Table 1 for terminology).

**Table 1:** Terminology related to evidence-based conservation guidelines (see text for further details)

Terms	Definitions
Evidence	Information (of varying strength; OCEBM Levels of Evidence Working Group 2016) to support a causal assertion on which a recommendation is based (e.g., National Institute for Health and Care Excellence 2014, p. 211).
Evidence-based conservation	→ Evidence-based practice in conservation (Pullin and Knight 2001, 2003).
Evidence-based guidelines	Regularly updated recommendations based on the best available evidence. Provides systematically collated, selected, assessed, and synthesized evidence together with systematically formulated recommendations of different strength. All steps are based on predefined, transparent methods. (e.g., Institute of Medicine 2011, pp. 4; National Institute for Health and Care Excellence 2014, p. 17) Supports → evidence-based practice. Synonym: Evidence-based medical guidelines (main text: medical guidelines).
Evidence-based management	→ Evidence-based practice in management (Walshe 2001).
Evidence-based medicine	Concept about “the conscientious, explicit, and judicious use of current best evidence in making decisions about the care of individual patients” (Sackett et al. 1996). Decisions integrate the best available evidence with clinical expertise and further aspects (e.g., patient preferences).
Evidence-based practice (EBP)	More general term for → evidence-based medicine, a concept which has spread into disciplines such as nursing and conservation (Pullin and Knight 2001; Mackey and Bassendowski 2017). Synonym: evidence-based decision-making.
Guidelines	Recommendations for practice. Differs from → evidence-based guidelines in that it lacks its characteristics (e.g., systematic evidence search, selection, and assessment) and the emphasis on evidence.
Manual	In the context of evidence-based practice, this is a handbook for the development of systematic reviews or evidence-based guidelines. Examples are the Cochrane Collaboration (2019) and National Institute for Health and Care Excellence (2014).
Meta-analysis	Quantitative method for the synthesis of the results of multiple studies (Deeks et al. 2019). Supports → evidence-based practice.
Systematic review	Method that systematically searches, selects, assesses, and synthesizes all available evidence to answer a specific question based on predefined, transparent methods (Collaboration for Environmental Evidence 2018; Sutherland and Wordley 2018; Chandler et al. 2019). Supports → evidence-based practice.

**Fig. 1:**
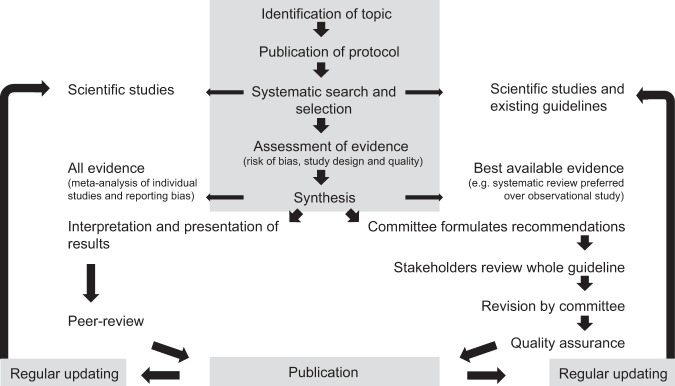
Outline showing the similarities (gray background) and steps to take (indicated by fat arrows; thin arrows: additional information) in a systematic review (left, center) and evidence-based guidelines (right, center). Adapted from National Institute for Health and Care Excellence (2014, p. 17); World Health Organization (2014); Cochrane Collaboration (2019)

Medical guidelines include informative and more normative parts (e.g., Sackett et al. 1996; note that we call a methods document on the preparation of evidence-based guidelines a manual, not a guideline). In both systematic reviews and medical guidelines, the scientific evidence is first systematically searched, selected, and then assessed based on predefined methods specified in a protocol (Fig. 1). The study design and quality (i.e., reliability) of individual studies can be expressed as levels of evidence (LoE), with LoE one indicating strong, and lower levels weaker evidence (e.g., OCEBM Oxford Centre for Evidence-Based Medicine Levels of Evidence Working Group 2016). Systematic reviews typically include all individual scientific studies (including weaker studies) and are themselves included in medical guidelines, which include only the strongest (best) available evidence. The synthesized evidence constitutes the informative part that is characteristic of both systematic reviews and medical guidelines (Fig. 1). Based on the best available evidence and practical aspects (such as patient preferences, utility-harm ratios, costs, and legal commitments), a committee including physicians, scientists, and patient groups formulates participatory process recommendations (e.g., Satterfield et al. 2009; World Health Organization 2014, pp. 69, pp. 123). Grades specify the strength of each recommendation (e.g., whether a medical treatment shall be conducted or better not). In the final medical guideline (which is reviewed by industry: World Health Organization 2014, pp. 69), recommendations of different strengths build the more normative part that is uncommon to systematic reviews (Fig. 1; e.g., GRADE Working Group 2004; Cochrane Collaboration 2019). Such final recommendations can be presented in a separate row to distinguish them from the gathered evidence. How exactly the recommendations in medical guidelines are based on the scientific evidence should be made transparent (Institute of Medicine 2011, pp. 75; National Institute for Health and Care Excellence 2014, pp. 161).

**Fig. 2:**
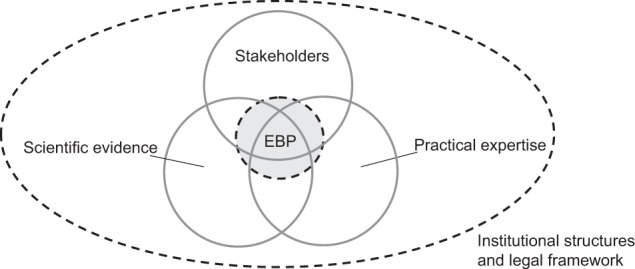
Evidence-based practice in biodiversity conservation integrates scientific evidence with the social dimension, practical expertise, and stakeholder interests related to multiple ecosystem services. Decisions (or policy outcomes) can be located in one, two, or all three circles. For example, in the absence of scientific evidence, recommendations can be based mainly on practical expertise. Figure adapted from Satterfield et al. (2009) © 2009 Milbank Memorial Fund with kind permission of Wiley

In ecology, established approaches toward evidence-based conservation include meta-analyses, systematic reviews, and synopses (Davies et al. 2006; Sutherland et al. 2017; Sandström et al. 2019). All approaches focus on evidence synthesis. Graded recommendations formulated as systematically and transparently as medical recommendations are lacking. Both scientific evidence and recommendations (which combine scientific evidence, stakeholder interests, and practical expertise) are part of evidence-based practice (Figs. 1 and 2). Expanding on existing approaches (such as systematic reviews), we propose that in forest conservation, the formulation of evidence-based guidelines would follow the steps outlined for medical guidelines (Fig. 1, from topic identification to publication). An evidence-assessment tool similar to those used in the medical field already exists for the ecological context (Mupepele et al. 2016). LoE created by this tool indicate evidence strength (i.e., study design and quality, Table 2), which is useful, for instance, for weighting individual studies included in meta-analyses (Mupepele and Dormann 2017) or weight of evidence frameworks (Suter and Cormier 2011; Collier et al. 2016). Formulating recommendations is a participatory process (similar to the medical field; e.g., Qaseem 2010, now including scientists, forest practitioners, and users). To date, the outlined steps characteristic of potential evidence-based guidelines are also uncommon in more comprehensive forest conservation guidelines, which typically lack one or more of the following: publication of a protocol, systematic evidence search and selection, standardized assessment of included evidence, grading of recommendations, full transparency of all methods, regular updating of publication (e.g., Ontario Ministry of Natural Resources 2010a, b; Humphrey and Bailey 2012; Spielmann et al. 2013; Wisconsin Department of Natural Resources 2013; Forestry Commission^2017^, pp. 43).

**Table 2:** Levels of evidence are essential to evidence-based guidelines as they indicate study strength. In contrast to evidence-based guidelines, levels of evidence are already available for the ecological context (Mupepele et al. 2016, © 2016 The Ecological Society of America with kind permission of Wiley)

Level of evidence	Description	Syntax
1	Literature review available (e.g., systematic review)	Very strong evidence
2	Study with control available (e.g., case–control study) or several lines of evidence with LoE 3	Strong evidence
3	Study without control available (study with inferential or descriptive statistics, e.g., histogram) or several lines of evidence with LoE 4	Moderate evidence
4	Expert opinion (no data available)	Weak evidence

The gap between science and practice in the field of ecology has been frequently described and discussed (e.g., Balmford and Cowling 2006; Haseltine 2006; Knight et al. 2008; Hill and Arnold 2012; Müller and Opgenoorth 2014). It has also been highlighted by many authors that more transparent and better structured translational approaches (which are common in evidence-based medical practice) could potentially narrow this gap and complement existing decision support tools (such as multi-criteria decision analysis, Acosta and Corral 2017; Pullin and Knight 2001; Sutherland et al. 2004; Schlesinger 2010; Sutherland and Wordley 2017). However, research indicating to what degree this potential could indeed be realized in ecology is rare. Most prominently, Dicks et al. (2014) show that for the case of the European Union’s Common Agricultural Policy, the evidence-based medicine framework would result in more effective policy outcomes. We expand upon the findings of Dicks et al. (2014) by arguing that an approach that integrates evidence with the social dimension (stakeholders and practical expertise, Fig. 1) could also facilitate the complex decision-making process in multiple-use landscapes such as forests. In this study, we explore the potential of evidence-based guidelines (introduced in Fig. 1 and Table 1) in forest biodiversity conservation.

Concerns about evidence-based practice (e.g., cookbook approach, evidence deficit, medical guideline complexity) are well studied in health care (e.g., Straus and McAlister 2000; Gibbs and Gambrill 2002; Bellamy et al. 2006; Sadeghi-Bazargani et al. 2014). For example, Bellamy et al. (2006) searched the literature for studies on evidence-based practice in mental health services and bolstered their identified concerns with eight expert interviews. There is a lack of analogous studies in forestry and forest conservation in particular (Pullin and Knight 2005). Accordingly, there is widespread ignorance of the efficiency of potential evidence-based forest guidelines. Opaque decision-making structures make it useful to study forest biodiversity conservation (e.g., dead-wood creation, Sutherland et al. 2017, pp. 144). Guidelines similar to the medical examples do not yet exist for this field of forestry (see, e.g., Center for International Forestry Research 2017). To better assess the chances of such novel guidelines in forest conservation, it is essential to know about potential problems that could arise from their application (i.e., during operational decision-making at the forest stand level) and development. The relationship between science and practice can be best improved by establishing more and closer links between scientists and practitioners (e.g., Roux et al. 2006; Petrokofsky et al. 2010). Therefore, we engaged directly with forest practitioners (state district foresters, regional-level administration, and private forest owners) to elicit concerns related to a potential evidence-based guideline that could increase the transparency of decision-making for the specific case of biodiversity conservation in forests. Our research objectives were to investigate (1) whether and which concerns are raised related to (2) the application and development of evidence-based guidelines in forest conservation. Similar to medical studies (e.g., Bellamy et al. 2006; Straus and McAlister 2000), we expected that more fundamental concerns would be raised mainly by forest practitioners. Furthermore, we aimed to discuss (3) the potential of such guidelines for the management of multiple-use landscapes by comparing identified concerns to challenges experienced by medical guidelines.

## Materials and Methods

Our sample of interviewees covered a broad range of professional functions that we expected to be involved in the application and development of potential evidence-based guidelines. The sample included six state district foresters, three persons working mainly in (or, e.g., as a consultant for) administration (regional council), three scientists doing more applied research and two private forest owners living and working in Germany, Switzerland, and Poland (one interview was conducted in English; see Table 3). Private forest owners and state district foresters were considered by us to be potential guideline applicants, as interviewees from both groups execute nature conservation measures planned by the forest administration (e.g., habitat tree selection, Spielmann et al. 2013, pp. 33). We were mainly interested in application (i.e., front-end) and development (i.e., back-end) related concerns (rather than in scientific methodology, e.g., evidence assessment, Mupepele et al. 2016). Note that state district foresters may also manage private forests or advice private forest owners. This overlap of professional categories allowed us to keep the sample size smaller than would have been necessary without such overlaps.

**Table 3:** Overview of interviewees’ main professional backgrounds, places of residence, language used in the interview, and sampling technique (p purposive, s snowball) used (sorted by profession)

Interview ID	Professional background	Country	Language of interview	Sampling technique
1.03.17.	Consultant	Germany	German	p
11.05.17.	Private forest owner	Germany	German	s
14.07.17.	Private forest owner	Germany	German	p
2.03.17.	Regional council	Germany	German	p
7.05.17.	Regional council	Germany	German	p
6.05.17.	Scientist	Germany	German	p
12.06.17.	Scientist	Switzerland	German	s
13.06.17.	Scientist	Switzerland	German	p
4.04.17.	State district forester	Germany	German	p
5.04.17.	State district forester	Germany	German	p
8.05.17.	State district forester	Germany	German	p
9.05.17.	State district forester	Germany	German	p
10.05.17.	State district forester	Germany	German	p
15.10.17.	State district forester	Poland	English	p

Note that the content of the column “Professional background” is simplified (e.g., the consultant had professional experience in applied forest science, consultancy, and was in contact with administration)

We used purposive and snowball sampling (which are often applied techniques in qualitative research, Miles and Huberman 1994, p. 27) to select 12 and 2 interviewees, respectively. Purposive sampling (i.e., sampling based on the judgment of the researcher) allowed us to select interviewees with a high level of experience and knowledge in their professional field. These interviewees were asked to provide the contact details of potential additional study participants; if those potential participants were included, we then asked them to provide contact details for additional participants (snowball sampling). The combination of the two sampling techniques allowed us to include two interviewees who we expected would have otherwise not participated (due to disinterest in the topic or limited time). In addition we were able to follow the principle of “inner representation” (Merkens 1997, p. 100), which aims to maximize heterogeneity within the sample of interviewees. For example, we had the chance to gather viewpoints from private forest owners with pronounced economic interest but also interviewed forest owners who managed their forests with a clear focus on biodiversity conservation. The main research areas among the scientists included in our sample ranged from the development of harvesting techniques to biodiversity conservation. The first contact with interview candidates was via e-mail (except for one case). In total, 22 interview candidates were contacted between January and October 2017; eight candidates did not reply.

**Fig. 3:**
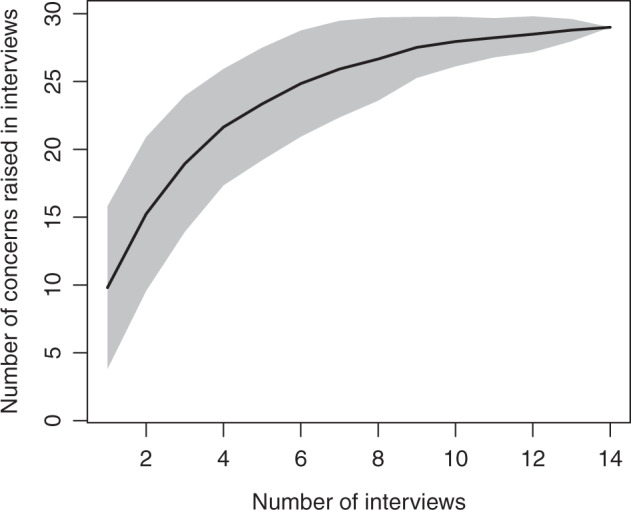
The curve (gray shaded: 95% confidence intervals) is asymptotic to the horizontal, suggesting that the majority of concerns were identified by a sample size of 14 interviews

The purposive sample size was defined by data saturation, which can occur in a sample with fewer than 12 participants for interviews conducted in different countries (Bertaux 1981, pp. 37; Guest et al. 2006). We computed the accumulation curve (specaccum, R package vegan, Oksanen et al. 2019) to assess the completeness of our sample. The curve indicates that we included the majority of concerns about evidence-based guidelines (Fig. 3).

We sent interviewees one A4-sized page with information about the research project attached to our e-mail interview request, which also included a short cover letter. In addition, all interviewees were offered a paper version of the same information leaflet, and the research project was explained by the interviewer if there was interest. We offered to visit interviewees at their workplace in a quiet room without other people. Two interviews took place in rooms provided by the University of Freiburg. All interviewees signed a current declaration for the collection and processing of interview data (Liebig et al. 2014). Ethical approval for this kind of research was not required but was still requested by the authors and received by the ethics committee Freiburg (application number: 10005/19, after study was conducted).

To illustrate the evidence-based approach, at the beginning of all interviews, excerpts of a medical guideline (Leitlinienprogramm Onkologie 2014b, pp. 1, pp. 141) were briefly presented, followed by evidence-based statements on forest conservation, specifically dead-wood retention and expected biodiversity response (hereinafter: statement paper; 1 page, Table 4 and Online Resource 1). The medical guideline was selected for its high level of quality (S3) and intuitive comprehensibility by a layperson.

**Table 4:** Exemplary evidence-based statements (adapted from Leitlinienprogramm Onkologie 2014b, pp. 141, and the statement paper) that were presented during the interviews

Grade of recommendation & Level of evidence	Recommendation
Grade of recommendation B (should)	Dermatoscopy should be conducted in cases of suspected diagnosis. It should be applied to enhance the clinical diagnosis of melanocytic lesions.
Level of evidence 2++	Kittler et al. (2002), Bafounta et al. (2001)
	Strength of consensus: 82%
Grade of recommendation A (shall)	The retention of 30 m^3^/ha of dead wood in European forests nevertheless results in a loss of habitat specialists but can conserve a wide range of species
Level of evidence 1	Gao et al. (2015), Lassauce et al. (2011), Müller and Bütler (2010), Paillet et al. (2010), Seibold et al. (2015)
	Strength of consensus: –

++ is used in this guideline for a finer subdivision between levels of evidence and represents a level between 1 and 2 (equivalent to high-quality systematic overviews). Within one level, all cited studies are of similar quality and design

Similar to the medical guidelines, in the statement paper, the confidence in the available literature was expressed as LoE ranging from one to four (Mupepele et al. 2016, Table 4). Based on this confidence, we formulated management recommendations (as was also done in the presented medical guidelines). Grades of recommendation included A (shall), B (should), and 0 (can; Leitlinienprogramm Onkologie 2014a, pp. 37). The recommendation for forestry is that 30 cubic meters (m^3^) per hectare (ha) of dead wood shall be retained in European forests. The statement in Table 4 was formulated to indicate that this is the absolute minimum retention. Strength of consensus does not apply to the dead-wood guidelines as no formal evidence-based guidelines were developed. The statement paper can only be a prototype of a potential guideline in forest conservation because it is currently unknown if the scheme is realistic in forest conservation. Our statement paper served as a conversation starter to show how the concept of evidence-based guidelines (illustrated by the example medical guidelines) could be applied to forest conservation.

The suitability of the questions in our interview guide (see Online Resource 2) to elicit the desired information was discussed among the researchers before pretesting them in two interviews. Studies from the medical field that were analogous to this one were read in full after all the interviews were completed to minimize priming effects (Tulving 1982). Questions were not provided in advance to the interviewees (as implied in, e.g., Kaiser 2014, pp. 51). After the initial presentation about evidence-based practice and apart from our guiding questions in between, the interviewees did the talking. At the end of the interviews (each of which took between ca. 40 and 115 min in total), we asked the interviewees whether they would like to add or emphasize particular points (as suggested in, e.g., Bogner et al. 2014, p. 61).

We rarely had the impression that interviewees withheld their concerns about evidence-based guidelines. To minimize the chance of missing a concern if necessary, we emphasized our neutrality as researchers, noted the anonymity of interviewees and used questions that explicitly aimed to trigger concerns.

After each interview, a postscript (including meta-data and a description of the interview atmosphere) and a memo (which summarized the main content) were written to help us remember the context of the interviews and interpret the content (e.g., Helfferich 2009, p. 193). Interviews and memos were linked with an ID to the postscripts and saved on a separate device in accordance with data privacy laws (e.g., Liebig et al. 2014). We did not receive any requests by interviewees asking for the provision of interview transcripts. No repeat interviews were conducted. All interviews were semistructured (Gill et al. 2008) and conducted by F.G.

**Fig. 4:**
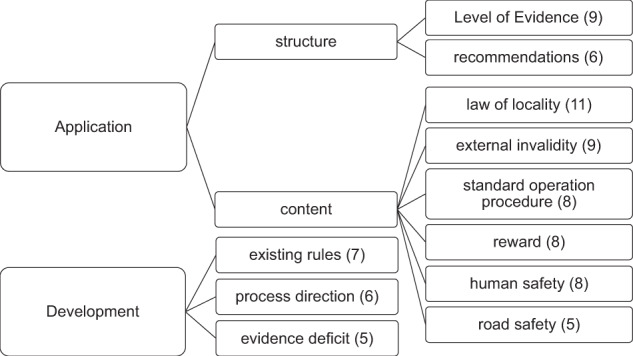
Codes linked to concerns mentioned frequently (five times or more) by interviewees. Codes are grouped depending on their relevance for application and development of potential guidelines. Numbers in parentheses indicate the number of interviews during which the concerns were raised. For further explanation of concerns, see text (or Table 5)

We employed inductive content analysis (Elo and Kyngäs 2008; Mayring 2000) as a method to gain an in-depth understanding of concerns related to potential evidence-based guidelines. Our analytical approach is particularly useful if the research topic is largely unexplored (Elo and Kyngäs 2008; Gill et al. 2008). After anonymisation (described in, e.g., Helfferich 2009, pp. 190; Liebig et al. 2014) and verbatim transcription, the text documents were coded by the same person. Coding was completed in MaxQDA (version 12.3.2, Verbi software, Berlin, Germany), allowing us to develop a coding tree (Fig. 4) and to identify the number of concerns mentioned by each interviewee. The kappa statistic was used to test the reliability of the code description (Cohen 1960). We randomly selected two transcripts for recoding and calculated the kappa coefficient in R on the basis of codes assigned/not assigned in each transcript (psych package, Revelle 2016). The resulting kappa coefficient of 0.71 confirms that the codes could be applied by a second person with good reliability.

## Results

We identified 28 concerns that could impede the application or development of potential evidence-based guidelines in forest conservation. Subsections are sorted by their frequency of occurrence during the interviews. The most frequently mentioned concerns are shown in Fig. 4 (see also Table 5: Overview of all concerns).

**Table 5:** Overview of all codes developed during inductive content analysis of interviews (sorted by number of interviews in which the code occurred)

Code	Description of concern	Occurrence (number of interviews)
Law of locality	Lack of specificity (e.g., missing specification of forest or soil types, climate), cookbook approach.	11
External invalidity	Assumed invalidity of scientific findings from other environmental conditions or lack of generality (e.g., range for dead-wood threshold).	9
LoE	Concerns founded in arguments against the LoE (e.g., visual appearance, categories used, complexity).	9
Reward	Lack of benefits (financial, social, or legal). “Social” refers to nonmonetary forms of valuation such as appreciation from the state (e.g., by the local district forester or a note in a newsletter) which could compensate for implemented conservation measures.	8
Standard operation procedure	Lack of detailed and easy to understand instructions how to best retain dead wood (possibly complemented by, e.g., pictures, examples or offers to take part in excursions, information about where to do what and when).	8
Human safety	Missing specifications regarding the safety of lumbermen working in and visitors of forests.	8
Existing rules	Best practice, laws, regulations, or guidelines that integrate evidence on dead-wood retention into practice (but lack a transparent evidence assessment, e.g., the old and dead-wood concept, Spielmann et al. 2013, pp. 33).	7
Recommendation	Concerns founded in arguments against the recommendations (e.g., visual appearance, categories used, complexity).	6
Process direction	A potential guideline that lacks approval of higher administrative levels (e.g., the state forest enterprise, ministry, local forest administrations, or the next level supervisor) will face low acceptance.	6
Evidence deficit	Lack of (reliable) evidence regarding the biodiversity-dead-wood links and resulting uncertainty in recommendations.	5
Road safety	Missing specifications regarding safety of transportation ways.	5
Ecological awareness	Existing awareness of guideline applicants (e.g., state district forester, private forest owner) about natural processes or environmental problems.	4
Harvesting access	Missing considerations of potential access limitations for harvesters posed by dead-wood retention.	4
Information overload	General reluctance (of decision makers or private forest owners) to process additional information about biodiversity issues in the form of elaborate (rather than short and precise) recommendations despite existing ecological awareness.	4
Point in time	Late involvement of interested groups during the development of a potential guideline	4
Surveillance	Lacking controls of the implementation of (publicly funded) dead-wood retention measures (which can be labor-intensive) would be a concern.	4
Dilution	Different parties (e.g., NGOs, politicians, scientists) negotiate possible recommendations based on the available evidence, and the result is a weak outcome, the lowest common denominator.	4
Interest conflict	One-sided maximization of (non-)monetary values by parties involved in the development of a potential guideline possibly resulting in societal disadvantages.	3
Involvement and appreciation	Lack of stakeholder involvement or consideration during the development of a potential guideline.	3
Goals	Lacking definition of what forest management should achieve if statement paper recommendations are followed.	3
Property right	Lacking consideration of forest ownership status (private, state, municipal).	2
Soil health	Lacking consideration of soil health.	2
Timber production	Lacking consideration of wood as a resource for the production of goods and services.	2
Lack of interest	Biodiversity-related topics have low priority on personal agenda of evidence-based guideline applicants (e.g., state district forester, private forest owner).	2
Rewarding structures	Complicated, time consuming, or nontransparent application (for funding of dead-wood retention), questionable financial incentives (which could, e.g., promote the use of heating oil instead of firewood and therefore should be avoided, according to an interviewee in favor of firewood production rather than dead-wood retention for biodiversity conservation); in particular, funding schemes by the European Union were sometimes perceived as being too complex and time consuming in relation to the received amount of money.	2
Consensus structures	Time consuming or complex administrative processes, which hinder the development of a potential guideline.	1
Formulation	Passive wording of biodiversity benefits from dead-wood retention (too much emphasis on knowledge gaps and uncertainty).	1
Traditions	Concerns founded in historical or emotional arguments (strong spiritual connection to own land, forest is passed on from generation to generation).	1

### The Scientist’s Dilemma of Providing Weak but Specific or Stronger but More General Advice

A majority of the interviewees (11) detected a lack of specificity in our presented statement paper about dead-wood retention. There was a wish for more detailed information regarding the influences of climate (season and altitude), forest types (deciduous, coniferous, mixed), and accessibility (slope, aspect) on how to retain dead wood. Furthermore, according to the majority of interviewees (eight persons), the instructions included in a potential guideline in forest conservation should be as detailed as possible. Similar to a standard operation procedure, such instructions preferably should include clearly defined management goals and state which forest operations should be carried out, as well as when, where, and how they should be carried out, to conserve biodiversity. The same persons who wished for more detailed descriptions about how scientific findings apply to their local context frequently also brought forward serious doubts about the external validity of studies conducted in other contexts (e.g., in the boreal biome) and sometimes questioned the acceptance of low recommendation grades (which can be a consequence of very specific recommendations). It was suggested in one interview that this poses a major challenge for a scientist who is asked for recommendations (citations of interviewees were translated from German, see Online Resource 3):

“… actually, one can only do it wrong. On the one hand, if one provides CLEAR numbers, which are already supported by the knowledge we have at the moment, it can be dangerous. On the other hand, if one does not provide them, this can also result in lacking acceptance [of evidence-based statements].” (I1)

The dilemma of giving evidence-based recommendations that also apply under particular circumstances was further illustrated by the same interviewee cited above, who suggested that it is difficult to convince everyone of the validity of the currently widely cited threshold of 30 m^3^ of dead wood per ha as long as rare saproxylic beetles (e.g., *Sinodendron cylindricum*) also occur on a table in the backyard with a few fruit trees and no other wood.

### Concerns Related to the Evidence Level

In almost 50% of the interviews, it was mentioned that the level of evidence is too complex as a measure of reliability. One interviewee stated that his own traditional knowledge and experience are placed too low in the evidence hierarchy. Three more persons hinted in the same direction by emphasizing the importance of knowledge gained through experience. One interviewee who was experienced in the formulation of management concepts expressed the concern that the burden of making the right management decisions is passed down to the lowest level (district forester) if LoEs are provided. Instead, people at higher levels (e.g., scientists) should take more responsibility in formulating recommendations that the end user can trust without being familiar with the LoE framework. Another interviewee who was experienced in the development of management concepts supported this:

“The practitioner [referring to state district foresters] says I need a short and precise instruction; the rest is not at all of interest for me. I don’t care if this is now LoE one or two. Besides, we know it better anyways. ... Terms like ‘level of evidence’, ‘grade of recommendation’, these are STRANGE words, these are completely strange terms, which you also had to arduously explain to me. Explain this to a thousand district foresters who are all saying, ‘Actually, I should urgently make timber now.’” (I2)

However, in general, forest practitioners (state district foresters, regional-level administration, and private forest owners) showed interest in scientific findings and in understanding why anecdotes not supported by representative data were placed low in the evidence hierarchy.

### Concerns Related to the Recommendations

Concerns related to forest multifunctionality (sensu, Borrass et al. 2017) that need to be considered in recommendations for a potential guideline were commonly mentioned (ten interviews). Among these, human safety in the forest and infrastructure maintenance were elaborated upon most frequently. For example, unstable standing dead wood was perceived as a major challenge, that affects several groups of people, most importantly lumbermen, foresters, hunters, and visitors (such as mountain bikers, mushroom pickers, and weekenders). It was pointed out that decaying or dead trees blocking infrastructure (e.g., roads, mountain bike trails, or cable railways) pose a high danger for all the aforementioned groups and that taking working security seriously can substantially limit regular harvesting activities.

Several interviewees also suggested taking into account the production function of forests, which would be reduced by standing or lying dead wood because of access limitations for large machines (e.g., harvesters). Furthermore, it was emphasized strongly in two interviews that dead wood retained in the forest is lost for timber production. It was illustrated by one interviewee that current handbooks and concepts often follow a segregated approach that emphasizes biodiversity conservation but neglects forest multifunctionality (and lacks practical instructions):

“The HANDLING of dead wood, this is NOT described. This means that the nature conservation aspect is usually well DESCRIBED, EVERYTHING. BUT, what am I doing with it and how do I PROCEED IF I HAVE THIS? Now the tree is standing at the trail and poses a danger. Do I harvest it, or how do I do that… There are no existing recommendations so far.” (I3)

In addition, the lack of compensation mechanisms, especially the importance of appreciation in terms of money, could limit the application of a potential guideline (eight interviews).

Recommendations were questioned in general (see, e.g., above, I2) in two interviews. Four interviewees raised concerns relating to visual appearance, categories used, and complexity. Furthermore, it was stated that most decision makers would suffer from information overload. Therefore, an intuitive presentation of the recommendations (similar to Table 4) was highlighted as essential.

### Further Concerns Brought Forward during the Interviews

In the following, we list concerns that we think are more difficult to address during the development of a potential guideline than the concerns listed before. Most prominently, existing legislation or concepts (which lack a transparent evidence assessment, e.g., the old and dead-wood concept “AuT,” Spielmann et al. 2013, pp. 33) were referred to as examples for integrating evidence into practice (seven interviews).

There exists an evidence deficit until scientists answer questions from practitioners. Such a deficit can limit trust in potential evidence-based guidelines. It may be that scientific evidence seemingly supports prevailing paradigms, which are overcome at a later point in time:

“Thirty years [ago], the forest hygiene was still different, right. Then, everything that was damaged was taken out. Now, it is different today.” (I4)

Another interviewee described how recent scientific findings (Schall et al. 2018) question the prevailing trend toward uneven-aged stands. These findings suggested that, depending on the scale of analysis, even-aged forest stands show higher diversity at the landscape scale of several species groups compared with uneven-aged stands (Schall et al. 2018). We consider the chronic evidence deficit as the root of the scientist’s dilemma described above.

### Overlap of Concerns between Professional Groups (Consultant, Private Forest Owners, Regional Council, Scientists, State District Foresters)

**Fig. 5:**
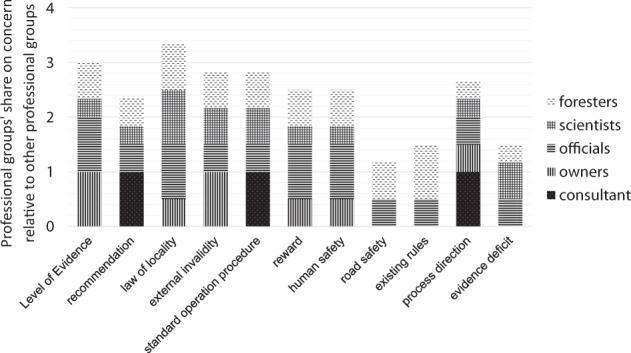
Most commonly mentioned concerns (Fig. 4) among each professional group (Table 3). Max. value is one per professional group and five per bar

Figure 5 shows percentages of interviewees who mentioned a common concern within each professional group (all data: see Online Resource 4). One scientist suggested that the structure of the LoE would be too complex for forest practitioners. For all interviewed scientists, the LoE was transparent and intelligible. Across most professional groups, there was a wish for more specific formulations regarding forest and soil type, organisms, and climate (law of locality). Similarly, concerns related to forest multifunctionality were a topic across most professional groups. However, the expression of multifunctionality related concerns may sometimes be a way to give timber production higher priority under more acceptable terms. The importance of incorporating safety aspects and considering existing frameworks related to dead-wood retention in potential evidence-based recommendations was noted in most interviews with state district foresters and mentioned by regional-level administration and private forest owners, but it was only briefly noted by one scientist.

## Discussion

### Forest Conservation and Medicine: Commonalities and Differences

In conservation management in general, few studies have been published about the limitations of evidence-based practice (e.g., Pullin and Knight 2005), but to the best of our knowledge no studies have addressed the challenges faced by evidence-based guidelines. This is different in the medical field, where literature reviews have examined concerns raised against evidence-based guidelines (e.g., Sadeghi-Bazargani et al. 2014).

**Fig. 6:**
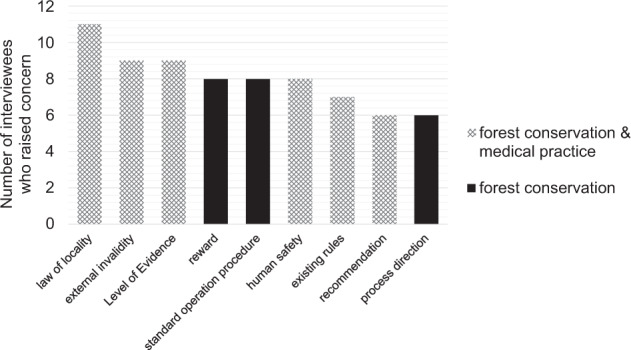
Bars with patterns indicate concerns mentioned frequently by interviewees (five times or more, as indicated on the *y*-axis) that were also found to be an obstacle in medical practice (based on Straus and McAlister 2000; Gibbs and Gambrill 2002; Sadeghi-Bazargani et al. 2014). Human safety is a concern related to preferred forest use. As medical equivalent, the patients’ values/preferences were selected. For further explanation of codes, see text (or Table 5)

Our study reports that forest conservation science faces a fundamental challenge. The scientist’s dilemma is that she can either provide weak but very specific or strong but general recommendations if evidence is limited. Neither will satisfy the advice-seeking practitioner. Concerns related to the applicability of general recommendations (similar to a cookbook of basic recipes) have also been described in the medical literature (e.g., Gibbs and Gambrill 2002; Fig. 6) and expounded as misperception of evidence-based practice (Straus and McAlister 2000). In addition, the root of the dilemma, which we see in a chronic evidence deficit, has been listed as a concern in reviews of the medical literature about evidence-based practice (e.g., Straus and McAlister 2000). It should be noted, however, that medical guidelines are often hundreds of pages thick, attempting to provide fast general and strong recommendations, before going into less supported but specific advice.

Our second main finding is that the structure of a potential guideline, in particular the evidence hierarchy, is at least initially too complex for the average forest practitioner (mainly state district foresters and private forest owners who, in contrast to physicians, are often not university graduates). The patient version of our example medical guideline limits itself to recommendations (Leitlinienprogramm Onkologie 2016). We can confirm that such a short version makes sense in forest conservation as well. The high complexity of evidence-based guidelines has also been mentioned as a concern in the medical field (Sadeghi-Bazargani et al. 2014; Fig. 6).

Our third main finding shows that the social dimension is often neglected in existing management concepts (but now increasingly addressed, e.g., Spielmann et al. 2013, pp. 33; Forestry Commission 2017, pp. 15), although the consideration of forest multifunctionality is essential, also for a high acceptance of potential guidelines. Interestingly, few interviewees specified the forest functions they were thinking of, beyond timber production and recreation (in connection with safety concerns). “Forest multifunctionality,” at least sometimes, seemed to be used as a euphemism for the economically important function of timber production.

In contrast to the studies from the medical field, the challenge of this study was to identify obstacles against yet-to-be developed evidence-based guidelines. The similarity in obstacles (e.g., cookbook approach, evidence deficit, evidence-based guideline complexity) between the two completely different scientific fields in retrospect justifies our methodology to present a statement paper and a medical guideline at the beginning of the interviews.

In addition to confirming common obstacles of evidence-based medical guidelines for forest conservation (Fig. 6), we also substantially extended the list of concerns. Most prominently, our results indicate a difference between forest conservation and medicine in that stakeholder interests are related to forest multifunctionality (i.e., integrated, partly conflicting goals such as conservation, production, and recreation). There is no doubt that similar goal conflicts exist in the medical field. For example, it has been suggested that the actual purpose of evidence-based practice is to reduce health care costs instead of increasing patient health (e.g., Gibbs and Gambrill 2002). However, in theory, the maintenance or rehabilitation of human health as an overall goal appears to be clearly defined.

A main argument against the use of medical guidelines was that it reduces professional freedom (e.g., Sadeghi-Bazargani et al. 2014). In contrast, the interviewees of our study often expressed the wish for more examples, illustrations and clearer instructions. Furthermore, the development and communication of evidence-based guidelines would be facilitated by following established hierarchical administrative structures (in Germany, starting at the state level down to local state district foresters or private forest owners, Fig. 6: process direction). This suggests that power structures in the professional environment and educational paradigms differ substantially between medicine and forestry.

### Towards Evidence-Based Guidelines: the Way Ahead

Based on our three main findings, we provide thoughts on how to present evidence-based guidelines for the management of multiple-use landscapes.

First, we suggest that the final guideline version for all practitioners (which we see as equivalent to medical doctors) should provide locally adapted recommendations that are written in plain language and intuitively structured (similar to Table 4). In contrast to many medical guidelines, these recommendations will have to be illustrated by examples and accompanied by more detailed instructions. In this regard, marteloscopes (1 ha forest sites used for exercising tree selection of inventoried trees: Schuck et al. 2016) could be useful as training sites. The wish for a diversity of presentation formats (detailed instructions, practical examples, and pictures) is in line with adjacent disciplines such as risk communication. Patients’ understanding of medical risk information was improved by visual aids (icon arrays, bar graphs) provided in addition to numbers (Zipkin et al. 2014) and numbers comunicated in addition to words (compared with the less preferred formats of just numbers or just words; Carey et al. 2018). Similarly, studies on environmental risk communication indicate that communication via different channels (television, radio) and formats (graphics, practical examples) is best practice (Höppner et al. 2010, pp. 53; National Oceanic and Atmospheric Administration 2016). In line with our results, plain language with clear instructions (and sometimes financial incentives to take action) is recommended over lengthy, abstract messages.

Second, practitioners should only be presented the recommendations (not the LoEs) to reduce complexity. However, to keep the development of evidence-based guidelines as transparent as possible, we suggest making the LoE (upon which the recommendations are based) available to the public in an open database.

Third, to better address the social dimension that brings in mainly landscape multifunctionality related concerns, we propose to first form a commission that is shielded from influential stakeholder interests. The commission could be part of a nonprofit organization (in the case of forests, e.g., the Forest Stewardship Council, the Programme for the Endorsement of Forest Certification Schemes, or the German Association of Forest Research Institutes), which takes responsibility for the development of evidence-based forest conservation guidelines. Akin to the formulation of medical guidelines, members of the commission (selected scientists and practitioners, such as state district foresters) would then present preformulated recommendations to a wider group of stakeholders (including politicians, representatives of the wood-processing industry) to assess their strength of support (indicated as consensus strength in Table 4).

It is a commonly underestimated strength of recommendations to account for various aspects, such as clinical experience and the patient’s will (Straus and McAlister 2000). We are therefore confident that such a commission would be an appropriate means to address concerns related to landscape multifunctionality more extensively than we could in our sample statement paper.

Last, recommendations should be regularly updated to include the best available evidence and adjusted based on their performance under local conditions. Such adjustments require long-term monitoring schemes.

## Conclusions

Evidence-based medical guidelines combine stakeholder interests, scientific evidence, and clinical experience in a structured and transparent process and therefore could potentially increase the effectiveness of decision- and policy-making in multiple-use landscape management. In the management of such landscapes, the concept of evidence-based guidelines suggests that evidence is integrated with the social dimension. The fact that our interviews highlighted this social dimension (despite being largely lacking in our exemplary statement paper) indicates that such guidelines have potential for the management of multifunctional landscapes. Further identified concerns about low specificity and high complexity were also described in medical studies. In contrast to medicine, we detected a wish of forest practitioners for more detailed but concise instructions. The emphasis on existing hierarchical structures that will frame the development of evidence-based guidelines and the high diversity of stakeholders were also unique to forest management. However, we did not find concerns that would make the development or application of evidence-based guidelines in the management of multiple-use landscapes fundamentally impossible. Therefore, we suggest conducting further research into the possibilities of this new evidence-based approach.

## Supplementary information

Online resource 1

Online resource 2

Online resource 3

Online resource 4
