# Supplementary material for: Exploration of Concerns about the Evidence-Based Guideline Approach in Conservation Management: Hints from Medical Practice
Source: Environ Manage. 2020 Jun 27;66(3):435–49. doi: 10.1007/s00267-020-01312-6 (PMC7434788; doi:10.1007/s00267-020-01312-6)
Supplement: Supplementary file 1 — Online resource 1 [file 267_2020_1312_MOESM1_ESM.docx]

**Online resource 1: Statement paper**

Preliminary statement about the efficacy of dead-wood retention for biodiversity conservation (statement paper)

1. Dependent variables: Species richness and abundance. These are influenced in which

way by

1.1 the dead-wood amount?

1.2 the spatial position of dead wood (horizontal, vertical)?

1.3 the dead-wood type (degree of decay)?

1.4 the dead-wood species?

1.1 Derivation of the Level of Evidence for **dead-wood amount**:

Several studies, including two meta-analyses^8, 9^ and three literature reviews^3, 5, 7^ have shown, that the dead-wood amount influences species richness^3, 5, 7, 8, 9^ and abundance^3, 9^ of saproxylic species. Müller and Bütler (2010) conclude, that dead-wood amounts below 30 m^3^/ha reduce the richness of saproxylic species in European deciduous forests. However, habitat specialists often require dead-wood amounts of >120 m^3^/ha^7^. An influence of the dead-wood amount on species that indirectly depend on dead wood has not yet been demonstrated clearly^9^.

Level of Evidence: **1**

Degree of recommendation:

The retention of dead-wood amounts of 30 m^3^/ha in European forests nevertheless results in a loss of habitat specialists but can conserve a wide range of species (**A**).

1.2 Derivation of the Level of Evidence for **spatial position** (horizontal, vertical) of dead wood:

There is evidence that increased sun exposure of dead wood can increase saproxylic species richness respectively abundance^9^. Results of an experimental study suggest that cavity nesters particularly benefit from dispersed snags if these are a limiting factor^4^. However, whether dead wood should be dispersed or clumped depends on the species and there has been done little experimental research on this subject^2, 4, 9^.

There is evidence that more species benefit from dead wood on the ground than from standing dead wood^9, 10^. However, results of several individual studies suggest that standing dead wood hosts habitat specialists (e.g. beetles of the genus *Germarostes*, *Rhyncolus* and *Stenoscelis*) which are rarely found on lying dead wood^9, 10^.

Level of Evidence: **2**

Degree of recommendation:

Sun exposed locations should be preferably used for dead wood retention (**B**). Based on the precautionary principle and weak evidence, standing as well as lying dead wood should be retained in dispersed and clumped distributions to provide habitat for a wide range of species (**B**).

Table A1: Degree of recommendation^1^

| Degree of recommendation | Description | Syntax |
| --- | --- | --- |
| A | strong recommendation | shall / shall not |
| B | recommendation | should / should not |
| 0 | recommendation open | can be considered / can be omitted |

Table A2: Level of Evidence^6^

| Level of Evidence | Description | Syntax |
| --- | --- | --- |
| 1 | Literature review available (e.g. systematic review) | very strong evidence |
| 2 | Study with control available (e.g. case-control-study)  **or** several lines of evidence with LoE 3 | strong evidence |
| 3 | Study without control available (study with inferential or descriptive statistics, e.g. histogram)  **or** several lines of evidence with LoE 4 | moderate evidence |
| 4 | Expert opinion (no data available) | weak evidence |

**Reference list**

1. Arbeitsgemeinschaft der Wissenschaftlichen Medizinischen Fachgesellschaften (AWMF)-

Ständige Kommission Leitlinien. AWMF-Regelwerk „Leitlinien“. 1. Auflage 2012.

http://www.awmf.org/leitlinien/awmf-regelwerk.html. Accessed 8 April 2020

2. Barton PS, Manning AD, Gibb H, Wood JT, Lindenmayer DB,

Cunningham SA (2011) Experimental reduction of native vertebrate grazing and addition

of logs benefit beetle diversity at multiple scales. J Appl Ecol 48(4):943–

951. https://doi.org/10.1111/j.1365-2664.2011.01994.x

3. Gao T, Nielsen AB, Hedblom, M (2015) Reviewing the strength of evidence of

biodiversity indicators for forest ecosystems in Europe. Ecol Indic 57:420–434.

doi: 10.1016/j.ecolind.2015.05.028

4. Kroll AJ, Duke SD, Hane ME, Johnson JR, Rochelle M, Betts MG,

Arnett EB (2012) Landscape composition influences avian colonization of experimentally

created snags. Biol Conserv 152:145–151.

https://doi.org/10.1016/j.biocon.2012.03.029

5. Lassauce A, Paillet Y, Jactel H, Bouget C (2011) Deadwood as a surrogate for

forest biodiversity: Meta-analysis of correlations between deadwood volume and species

richness of saproxylic organisms. Ecol Indic 11(5):1027–1039.

https://doi.org/10.1016/j.ecolind.2011.02.004

6. Mupepele AC, Walsh JC, Sutherland WJ and Dormann CF (2016) An evidence

assessment tool for ecosystem services and conservation studies. Ecol Appl

26(5):1295–1301. doi:10.1890/15-0595

7. Müller J, Bütler R (2010) A review of habitat thresholds for dead wood: a baseline for

management recommendations in European forests. Eur J Forest Res

129(6):981–992. https://doi.org/10.1007/s10342-010-0400-5

8. Paillet YL, Bergès J, Hjältén P, Òdor C, Avon M, Bernhardt-Romermann RJ,

Bijlsma L, De Bruyn M, Fuhr U, Grandin R, Kanka L, Lundin S, Luque T, Magura

S, Matesanz I, Meszaros MT, Sebastià W, Schmidt T, Standovar B, Tothmeresz A,

Uotila F, Valladares K, Vellak R, Virtanen (2010) Biodiversity differences between

managed and unmanaged forests: meta-analysis of species richness in Europe. Conserv

Biol 24:101– 112. https://doi.org/10.1111/j.1523-1739.2009.01399.x

9. Seibold S, Bässler C, Brandl R, Gossner MM, Thorn S, Ulyshen MD, Müller J (2015)

Experimental studies of dead-wood biodiversity — A review identifying global gaps

in knowledge. Biol Conserv 191:139–149.

https://doi.org/10.1016/j.biocon.2015.06.006

10. Ulyshen MD, Hanula JL (2009) Habitat associations of saproxylic beetles in the

southeastern United States: a comparison of forest types, tree species and wood postures.

Forest Ecol Manag 257(2):653–664.

https://doi.org/10.1016/j.foreco.2008.09.047
