# Supplementary material for: Exploration of Concerns about the Evidence-Based Guideline Approach in Conservation Management: Hints from Medical Practice
Source: Environ Manage. 2020 Jun 27;66(3):435–49. doi: 10.1007/s00267-020-01312-6 (PMC7434788; doi:10.1007/s00267-020-01312-6)
Supplement: Supplementary file 2 — Online resource 2 [file 267_2020_1312_MOESM2_ESM.docx]

**Online resource 2: Interview guide**

Questions to identify concerns ranging from application to development of potential evidence-based guidelines (interview guide)

Question types:

1) Example: 1. Main question, low presupposition

2) Example: 1.1 Follow-up question, more specific

3) Example: [keyword, very specific]

1. Please describe, how typical tasks that your current position requires look like. I think this could facilitate the following conversation.

2. Which thoughts do you have when reading the statement paper?

2.1 In the medical field guidelines are well established. But with regard to forestry this is

maybe not a good approach? What do you think?

3. Do you see a connection / relation between your current position and evidence-based guidelines?

**Application related concerns**

1. Aren't there already enough guidelines / concepts for the implementation of nature conservation in forest management? [Old- and dead wood concept, Spielmann, 2013, pp. 33, Natura 2000, PEFC- & FSC-certification schemes]
   1. Do you think that a statement paper offers an added value?
   2. Please describe how you would use a statement paper in your current work.
   3. Do you know colleagues who could make use of such a statement paper?
   4. How could a statement paper facilitate your decision making?
2. How would you design a statement paper so that it offers an added value?
   1. Do you have recommendations for the structure of the statement paper?

[LoE, recommendation grades, traffic light?, pictogram?, habitat requirements vs. intervention,

structural parameters, scientific results, traditional knowledge, external validity]

**Development related concerns**

1. Please imagine, you have the task to develop a guideline. How would you proceed? Possibly you can describe your course of action on the basis of an already implemented concept.
   1. For which occupational groups would you formulate such a guideline?
   2. Who could be the user of such a guideline?

[forest owners, lumbermen, forest rangers, administration (ministry for rural areas and consumer

protection of the federal state Baden-Württemberg, ForstBW, regional council, forestry authority)

- 1. Please describe, whether a categorization of studies based on their statistical design makes sense to you.
  2. Who would you commission to lead the development of a statement paper?
  3. Which stakeholders would you involve in the formulation of a guideline? [see above, forest owners, etc.]
  4. Please describe how different institutions and stakeholders could be involved.
  5. Do you see difficulties when different institutions and stakeholders are involved?
  6. How would you ensure that a guideline is applied?

**Next steps**

1. Is there a question / issue related to your occupation that you would like to have answered with a statement paper / literature review?
2. Do you know people from the forestry sector that would be interested in an interview?

**Short info about interviewee**

1. Are you willing to tell me what you like about your occupation and what not so much?
2. Are you working together with scientists?

10.1 How do you think about the exchange with scientists?

1. What are three important information sources that you use to stay updated in your field? Please rank according to importance. [Scientific studies?]

12. Last question: "All in all – do you have the impression, that we have missed to address aspects that are important from your point of view? Do you have something to add?" (Question recommended in Bogner et al. 2014, p. 61)

13. Call back: In case of further questions, it is possible to approach you via email or phone? (Question recommended in Kaiser, 2014, pp. 66)

**Reference list**

Bogner A, Littig B, Menz W (2014) Interviews mit Experten - Eine praxisorientierte Einführung. Springer Fachmedien, Wiesbaden (Germany)

Kaiser R (2014) Qualitative Experteninterviews - Konzeptionelle Grundlagen und praktische Durchführung. Springer Fachmedien, Wiesbaden (Germany)

Spielmann M, Bücking W, Quadt V, Krumm F (2013) Integration of nature protection in forest policy in Baden-Württemberg (Germany). INTEGRATE country report. EFICENT-OEF, Freiburg. http://www.efi.int/sites/default/files/files/publication- bank/projects/badenwuerttemberg.pdf. Accessed 15 October 2019
