# Supplementary material for: Exploration of Concerns about the Evidence-Based Guideline Approach in Conservation Management: Hints from Medical Practice
Source: Environ Manage. 2020 Jun 27;66(3):435–49. doi: 10.1007/s00267-020-01312-6 (PMC7434788; doi:10.1007/s00267-020-01312-6)
Supplement: Supplementary file 3 — Online resource 3 [file 267_2020_1312_MOESM3_ESM.docx]

**Online resource 3: Citations**

Statements of interviewees as transcribed

(Interview 1)

"...man kann's eigentlich nur falsch machen sowohl wenn man (.) KLARE (.) zahlen liefert die [I: ja] die schon auf den kenntnissen beruhen die wir im moment haben kann's gefährlich sein auf der anderen seite wenn man sie nicht liefert kann's auch zu fehlender akzeptanz führen"

(Interview 2)

"...der praktiker sagt einfach auch ich brauch 'ne kurze klare anweisung (--) [I: hm] des außen rum interessiert mich überhaupt net (.) ob des jetzt loe 1 oder e 2 isd is mir wurscht (.) außerdem wiss ma des eh alles selber besser (--) [I: hm]"

"also begriffe wie evidenzstärke (.) empfehlungsgrad des sind FREMDE wörter (.) [I: ok] des sind völlig (.) fremde begriffe die du mir ja jetzt auch (--) ähm mühsam erklärt hast [I: ja] erklär des mal (.) tausend revierleitern (--) [I: hm ok] ((leises lachen)) die alle sagen ich sollte jetzt eigentlich dringend holz machen ((beide lachen)) [I: ja]"

(Interview 3)

"...der UMGANG mit dem totholz (--) der isch (--) NICHT beschrieben [I: ja] ja des heißt der natur- äh der naturaschpekt der naturschutzrechtliche aschpekt isch in der regel gut BESCHRIEBEN (.) ALLES [I: ja] ABER (.) was mach ich damit (.) ja und wie geh ich VOR WENN ICH DES HABE [I: mhm] ja (.) und der baum steht jetz am weg und stellt 'ne gefahr dar (.) des fälle oder wie wie wie wie mach ich des (.) ich glaub des sind solche dinge äh die die dene da gibt's keine handl- bisher nicht ich glaub da sind sie auf'm richtige weg äh keine echte handlungsanweisung"

(Interview 4)

"...dreißig jahr war die waldhygiene noch anderschd ned da hät ma alles rausg'holt was kaputt war und ned und [I: ja] gepflegt und des ned (.) jetz isch es heut anderschd..."
