# Supplementary material for: Exploration of Concerns about the Evidence-Based Guideline Approach in Conservation Management: Hints from Medical Practice
Source: Environ Manage. 2020 Jun 27;66(3):435–49. doi: 10.1007/s00267-020-01312-6 (PMC7434788; doi:10.1007/s00267-020-01312-6)
Supplement: Supplementary file 4 — Online resource 4 [file 267_2020_1312_MOESM4_ESM.docx]

| **Code** | **consultant** | **owners** | **officials** | **scientists** | **foresters** |
| --- | --- | --- | --- | --- | --- |
| law of locality | 0.00 | 0.50 | 1.00 | 1.00 | 0.83 |
| external invalidity | 0.00 | 1.00 | 0.50 | 0.67 | 0.67 |
| LoE | 0.00 | 1.00 | 1.00 | 0.33 | 0.67 |
| reward | 0.00 | 0.50 | 1.00 | 0.33 | 0.67 |
| standard operation procedure | 1.00 | 0.00 | 0.50 | 0.67 | 0.67 |
| human safety | 0.00 | 0.50 | 1.00 | 0.33 | 0.67 |
| existing rules | 0.00 | 0.00 | 0.50 | 0.00 | 1.00 |
| recommendation | 1.00 | 0.00 | 0.50 | 0.33 | 0.50 |
| process direction | 1.00 | 0.50 | 0.50 | 0.33 | 0.33 |
| evidence deficit | 0.00 | 0.00 | 0.50 | 0.67 | 0.33 |
| road safety | 0.00 | 0.00 | 0.50 | 0.00 | 0.67 |
| ecological awareness | 0.00 | 0.50 | 0.00 | 0.00 | 0.50 |
| harvesting access | 0.00 | 0.00 | 0.00 | 0.33 | 0.50 |
| information overload | 0.00 | 0.50 | 1.00 | 0.33 | 0.00 |
| point in time | 1.00 | 0.00 | 0.50 | 0.33 | 0.17 |
| surveillance | 0.00 | 0.50 | 0.50 | 0.00 | 0.33 |
| dilution | 0.00 | 0.00 | 0.50 | 0.33 | 0.33 |
| interest conflict | 0.00 | 0.00 | 0.50 | 0.67 | 0.00 |
| involvement & appreciation | 0.00 | 0.50 | 0.50 | 0.00 | 0.17 |
| goals | 0.00 | 0.00 | 0.00 | 0.67 | 0.17 |
| property rights | 0.00 | 0.50 | 0.00 | 0.33 | 0.00 |
| soil health | 0.00 | 0.00 | 0.00 | 0.33 | 0.17 |
| timber production | 0.00 | 0.50 | 0.00 | 0.33 | 0.00 |
| lack of interest | 0.00 | 0.50 | 0.00 | 0.00 | 0.17 |
| rewarding structures | 0.00 | 0.50 | 0.00 | 0.00 | 0.17 |
| concensus structures | 0.00 | 0.00 | 0.50 | 0.00 | 0.00 |
| formulation | 0.00 | 0.00 | 0.00 | 0.33 | 0.00 |
| traditions | 0.00 | 0.50 | 0.00 | 0.00 | 0.00 |

**Online resource 4:** Data supporting Fig. 5
